# Using virtual reality to treat aggressive behavior problems in children: A feasibility study

**DOI:** 10.1177/13591045211026160

**Published:** 2021-06-20

**Authors:** Sophie C Alsem, Anouk van Dijk, Esmée E Verhulp, Bram O De Castro

**Affiliations:** 1 Department of Developmental Psychology, 8125 Utrecht University, The Netherlands; 2 Research Institute of Child Development and Education, 1234 University of Amsterdam, The Netherlands

**Keywords:** Cognitive behavioral therapy, aggression, virtual reality, children, feasibility, intervention

## Abstract

Evidence-based cognitive behavioral therapies (CBTs) for children with aggressive behavior problems have only modest effects. Research is needed into new methods to enhance CBT effectiveness. The aims of the present study were to (1) examine whether interactive virtual reality is a feasible treatment method for children with aggressive behavior problems; (2) investigate children’s appreciation of the method; and (3) explore whether children’s aggression decreased during the ten-session treatment. Six boys (8–12 years) participated at two clinical centers in the Netherlands. Newly developed weekly reports were collected on treatment feasibility (therapist-report), treatment appreciation (child report), and children’s aggression (child/parent report). Results supported treatment feasibility: therapists delivered on average 98% of the session content, provided more than the recommended practice time in virtual reality, experienced few technical issues, and were satisfied with their treatment delivery. Children highly appreciated the treatment. Parents reported decreases in children’s aggression over the treatment period (i.e., between week 1 and week 10), but children did not. The promising findings of this feasibility study warrant randomized controlled trials to determine whether interactive virtual reality enhances CBT effectiveness for children with aggressive behavior problems.

## Introduction

Aggressive behavior problems are among the most prevalent problems affecting children (Lochman & Matthys, 2017). Children with aggressive behavior problems are at heightened risk for adverse outcomes later in life, such as lower educational achievement, delinquency, substance abuse, and mental health issues (Burkey et al., 2018; Loeber & Farrington, 2000). To prevent escalation of aggressive behavior into persistent negative outcomes, it is important to treat aggressive behavior problems as they arise in childhood (Lochman & Matthys, 2017). Cognitive behavioral therapy (CBT) has been shown to reduce aggression in children (Weisz & Kazdin, 2017). However, effects have been modest and heterogeneous (McCart et al., 2006), with stronger effects for CBT interventions in which children practice more with anger exposure and solving real-life social problems (De Mooij et al., 2020; Landenberger & Lipsey, 2005). Therefore, new methods are needed that may enhance ecologically valid exposure and practice (Weisz et al., 2019). Interactive virtual reality is a promising method to enhance effects of current CBT for children with aggressive behavior problems. The present study investigated the feasibility of this treatment method.

Interactive virtual reality seems particularly beneficial to reduce reactive aggression—the most prevalent form of aggression in middle childhood (Vitaro et al., 2002). Reactive aggression can be defined as aggression in response to perceived threat or frustration (Dodge, 1990). To reduce this form of aggression, most CBT interventions target deficits in emotion regulation and social cognitive processing—two mechanisms underlying childhood aggression (Crick & Dodge, 1994; Lochman & Matthys, 2017). As part of CBT, children learn to monitor their anger and use techniques to modulate elevated levels of anger during social interactions (Chorpita & Daleiden, 2009; Sukhodolsky et al., 2016). This is based on the assumption that children’s aggression will decrease when they become better at regulating their anger. Therapists typically teach anger regulation in role plays (Garland et al., 2008; Sukhodolsky et al., 2016). In fact, role play practice is integrated in 83% of evidence-based child CBT and parent training interventions for children with externalizing behavior problems (Menting et al., 2015). Interactive virtual reality offers a virtual environment for role play practice. This method seems promising for three reasons: (1) it allows for individually tailored exercises in social interactions; (2) it provides an immersive and emotionally involving environment needed to practice emotion regulation; and (3) it may be a motivating and engaging treatment approach for today’s youth.

### Individually tailored exercises

Most CBT’s for children with aggressive behavior problems are delivered in group format, providing a natural context to practice in role play (Lochman et al., 2019). However, group formats may actually reduce CBT effectiveness. Prior studies have shown iatrogenic effects of group therapy for youth with aggressive behavior problems (‘deviancy training’; Dodge et al., 2006). Furthermore, the specific situations, cognitions, and behaviors that children need to practice in therapy will be unique to each individual child. Indeed, research has shown that individual therapy leads to larger decreases in children’s aggressive behavior problems than group-format therapy (Lochman et al., 2015; Wilson & Lipsey, 2007). Thus, “tailoring” treatment to each child’s needs and characteristics may increase CBT effectiveness—an advantage also recognized by therapists working with these children (Lochman et al., 2017b).

How then, can we provide the paradoxical combination of tailored individual treatment together with ecologically valid practice with actual peers? Interactive virtual reality may provide a solution. In interactive virtual reality, children can interact, talk, and play with virtual peers in different situations, controlled by the therapist. This allows them to practice new cognitions and behavior in tailored “role plays”—even though they receive individual treatment.

### An emotionally involving practice environment

Research suggests that therapy is most effective when cognitions and skills are challenged and practiced in emotionally involving situations (Suveg et al., 2007). Thus, children with aggressive behavior problems should ideally practice in anger-provoking situations (e.g., being provoked by peers; Sukhodolsky et al., 2016). Interactive virtual reality offers this possibility. First, interactive virtual reality can simulate anger-provoking situations encountered in daily life. In virtual reality, children are fully immersed in the virtual environment and do not have to rely on their memory or imagination to practice with these situations (Park et al., 2011). Second, virtual reality allows children to practice with dynamic, realistic social challenges, adapted to their abilities (Kandalaft et al., 2013). Therapists can trigger children’s anger within ethical boundaries, using a fully controlled virtual environment that can be adapted or stopped at any time. Third, virtual reality allows for repetitive practice in a stimulating environment (Newbutt et al., 2016; Saiano et al., 2015), allowing children to automatize newly learned skills. Indeed, a recent study showed that it is possible to repeatedly elicit children’s anger in virtual reality, so that they can practice repeatedly while remaining emotionally engaged (Verhoef et al., 2021).

### Motivating and engaging treatment approach

Children with aggressive behavior problems often display low motivation and resistance to treatment (Frick, 2012; Lochman et al., 2019). It is important to enhance children’s treatment motivation, which has been related to increase in treatment effectiveness (Lochman et al., 2017b). Virtual reality may serve this goal. It may appeal to children as it resonates with their involvement in digital innovations nowadays (Weisz et al., 2019). Indeed, using technology (i.e., adding an internet component) can increase treatment engagement of children with aggressive behavior problems (Lochman et al., 2017a). Similarly, virtual reality has been shown to enhance treatment motivation and attendance in adolescents and adults (Hadley et al., 2019; Park et al., 2011). Last, children with aggressive behavior problems who participated in virtual reality research indicated that they found this method very appealing (Verhoef et al., 2021).

### The present study

Given the potential advantages of using virtual reality, we decided to develop an individual CBT with interactive virtual reality for children with aggressive behavior problems. To our knowledige, this is the first time that virtual reality is implemented in therapy for children with aggressive behavior problems. Hence, we decided to conduct a small-scale feasibility study. The aim of this study was to describe the content, appreciation, and feasibility of this treatment approach. We developed the CBT “YourSkills” and designed a virtual reality–based version of this treatment, in which children practice emotion regulation and social information processing in interactive virtual reality to decrease their aggressive behavior. We examined the feasibility of YourSkills with virtual reality using therapist reports, investigated children’s appreciation of the treatment, and explored whether children’s aggressive behavior problems decreased during treatment according to both parents and children.

## Method

### Participants

Six boys referred for aggressive behavior problems were recruited at two clinical centers in the Netherlands. These centers provide mental health care for children with a broad range of mental health problems, including aggressive behavior problems. All children met the inclusion criteria for YourSkills: age (according to the clinic's records) 8–12 years, aggressive behavior problems, intelligence level above 80, no severe autism spectrum disorder, and no epilepsy or severe visual or auditory limitations. We chose to only include children from 8–12 years as these children are old enough to profit from CBT (McCart et al., 2006) and still in Dutch elementary school. Only boys were included because we initially developed YourSkills specifically for this group, considering that girls’ development, forms, and outcomes of aggression are found to be different from boys (Underwood, 2002). Therapists invited parents of children who met the inclusion criteria to participate in this feasibility study. Parents provided active consent for participation in this study. The study was approved by the Medical Ethics Committee of the Utrecht University Medical Center (NL67139.041.18).

### Procedure

This feasibility study followed children participating in ten weekly treatment sessions of YourSkills with interactive virtual reality. Therapists rated a short questionnaire after each session, assessing treatment feasibility. After the treatment, therapists indicated their satisfaction with treatment delivery and answered an open-ended question about their general evaluation of YourSkills. Children and parents filled out a short questionnaire during each treatment session, assessing children’s appreciation of the treatment (child report) and aggressive behavior (child and parent report) from pretreatment (week 1) to posttreatment (week 10).

### Treatment

YourSkills with interactive virtual reality is a manualized CBT, developed through iterative discussions between experienced healthcare psychologists and researchers. YourSkills is based on evidence-based treatments for children with aggressive behavior problems, including coping power (Lochman et al., 2008) and self-control (Van Manen, 2001). We chose to develop a new treatment manual, rather than adding virtual reality to an existing treatment. This way, we could integrate interactive virtual reality into all facets of our treatment (rather than merely replacing role play exercises with virtual reality).

YourSkills consists of one 45-minute introduction session with parents and ten 45-minute sessions with the child (Table 1). The aim of YourSkills is to reduce children’s aggressive behavior problems by enhancing emotion regulation and social information processing skills. Children practice anger recognition, anger regulation, and social problem solving in virtual social interactions. In each session, therapists first explain a new skill, then model the skill using role play, and then use virtual reality to let children practice the skill in anger-provoking social situations. YourSkills includes a reward system to motivate children to practice the newly learned skills. Children receive tokens for each time they practiced, both during the session and at home.

**Table 1.** Content of the YourSkills treatment sessions.

Session	Content^a^
Introduction session with parents	Parents learn about the content of the therapy
	Therapist emphasizes the importance of parent and teacher involvement (e.g. assisting in home work and reinforcing practiced skills at home)
	Parents formulate relevant anger-provoking situations for their child
1. Know yourself	Child and therapist get to know each other, and discuss the child’s strengths
	Therapist introduces emotions and explains how angry feelings link to specific situations
	Child becomes acquainted with interacting in virtual reality
	Child learns how the reward system and at-home training work
2. Check your feelings	Therapist explains how anger builds up, which is visualized using an anger stairs (similar to thermometer or stoplight methods)
	Child practices with recognizing angry feelings in virtual reality, focusing on bodily sensations and cognitions
3. Take a break	Child learns how to take a time-out (i.e., behavioral distraction) and practices this skill in virtual reality
4. Feel relaxed	Child learns how to use relaxation exercises (i.e., behavioral relaxation) and practices this skill in virtual reality
5. Think strong	Child learns how to use helping thoughts (i.e., cognitive reappraisal) and practices this skill in virtual reality
6. Think again	Child learns how to change negative interpretations (i.e., attribution retraining and perspective taking) and practices this skill in virtual reality
7. Make a smart move	Child learns that being agreeable can be the best solution (i.e., problem solving) and practices this skill in virtual reality
8. Stand together	Child learns how to ask an adult for help (i.e., problem-solving) and practices this skill in virtual reality
9. Pro training	Child rehearses the learned skills in virtual reality
	Child and parents agree on a “secret signal” as a reminder for the child
10. Expert training	Child rehearses the learned skills in virtual reality
	Child and therapist make a relapse prevention plan
	Parents, child, and therapist end the treatment with a certificate

^a^Additionally, all sessions start with an outcome rating scale and a discussion of last week’s homework and end with a session rating scale and a discussion of next week’s homework with parents.

All treatment sessions have the same structure, providing a predictable course of the sessions for children. First, therapists show children the session’s agenda and ask them to rate a brief outcome rating scale (see Measures). Next, therapists briefly discuss last week’s “at home training” (i.e., homework assignments, such as practicing relaxation at least three times). Most of the session time is then spent on practicing the session’s new skill in virtual reality. Children practice the same skill several times in different situations or with increasing difficulty level. After practicing, children rate a brief session rating scale to assess their appreciation of the treatment (see Measures). During the last ten minutes of each session, therapists invite parents, summarize the session, and discuss next week’s homework assignment.

Although YourSkills is primarily focused on the child, it also promotes parent involvement. Parents have an important role in child CBT: they are the ones to bring their child to the sessions, provide information about their child’s behavior, and recognize and reward their child’s efforts at home (Sukhodolsky et al., 2016). For this reason, YourSkills starts with an introduction session for parents (Table 1) and involves parents at the end of each session. Additionally, therapists send parents an email summarizing each session directly after each session.

In this study, YourSkills was delivered by four licensed therapists with a background in child psychology and CBT. Therapists received a two-day course in YourSkills, supervised by the first and second author and a certified psychologist. Therapists learned to work with the treatment manual and virtual reality equipment during the course and could receive consultation by phone during the treatment period.

### Virtual reality

**Figure 1.**
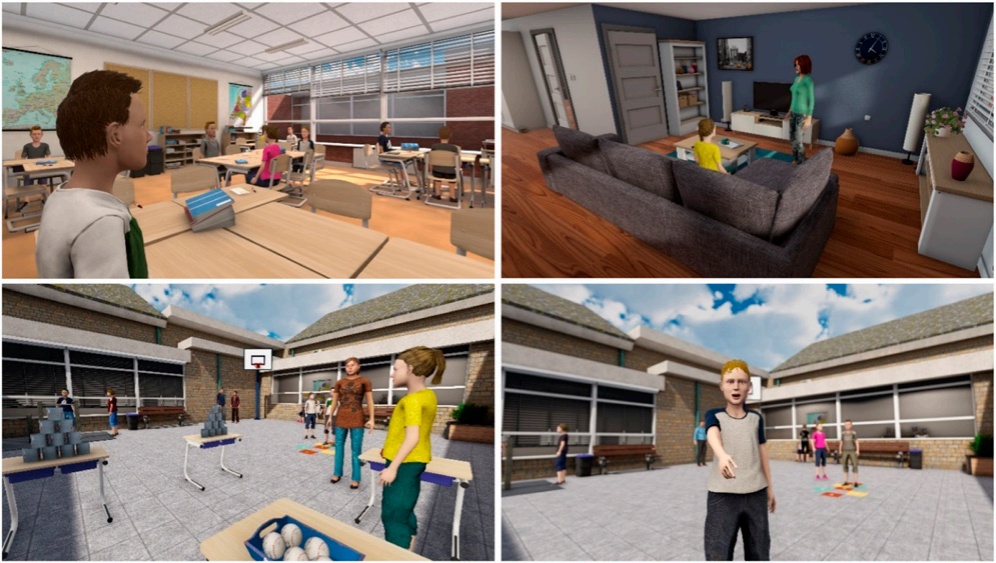
Virtual reality classroom, living room, and schoolyard environments.

The YourSkills virtual reality environment consists of a classroom, a schoolyard, and a living room, built by the technological company CleVR (see Figure 1). Children enter an immersive digital environment, where they have full mobility: they can walk around in the digital world, move objects, play games, and interact with virtual others. Children wore an Acer Windows Mixed Reality headset, a noise canceling headphone, and they held controllers in both hands, allowing them to grab and throw virtual objects. In the first session, therapists explained to children that the virtual environment allowed them to walk around freely (within a 3 × 3 m area), talk with virtual children and adults, and play games such as building a tower or playing a game on the television. Virtual peers were boys and girls from the same age range, who had average height and dark hair. Virtual adults were male and female characters with diverse physiques. Children were asked to select the characters that most resembled their parents, siblings, or teachers.

Therapists used the virtual environment to create challenging social situations for children to practice with. The YourSkills virtual reality software consists of twenty-six anger-provoking starting situations that therapists could tailor to children’s individual needs. These situations were based on a taxonomy of problematic situations for children with aggressive behavior problems. They include: being disadvantaged, authority conflicts, peer rejection, and peer provocation (Matthys et al., 2001). All situations were designed to trigger moderate levels of anger, aiming to help children practice their regulation skills while being emotionally engaged. Therapists fully controlled the virtual environment and could immediately adapt or stop the situation if needed.

Each session, therapists prepared children by explaining that they would use the virtual reality environment to try to evoke their angry feelings. Therapists could evoke children’s anger by manipulating the virtual situation itself (e.g., letting the child lose a game and switching off the television) or by manipulating the speech and actions of the virtual characters. Therapists used a microphone with voice transformer to emulate a different voice for each virtual character. They used a tablet to control the characters’ bodily movements (e.g., walking away), gestures (e.g., raising a middle finger), and facial expressions (i.e., an expression scale from happy to angry). The dynamic nature of virtual reality allows the therapist to tailor exercises to the specific needs and goals of the child, for example, by making provocative behavior of virtual peers more or less subtle, or by responding to children’s behavior in ways that would trigger their anger in daily life.

### Measures

#### Feasibility

Therapists rated four aspects of treatment feasibility after each session. First, they indicated whether they had completed each session element (e.g., discuss homework and explain skill) by rating “*done”* or “*not done.”* To estimate feasibility, we calculated the percentage of completed elements out of all ten elements in each session. Second, therapists reported any technical issues with the virtual reality equipment. Third, they estimated the time children practiced in virtual reality (recommended time was ten minutes). Fourth, they reported how many times children practiced a skill in virtual reality (recommended number was at least two times). After the last session, therapists completed three items on their satisfaction with treatment delivery across all sessions (i.e., “*For this child I successfully delivered the 10 sessions of the treatment,”* “*For this child I am satisfied with how I delivered the treatment,”* and “*For this child I am confident that I successfully delivered the treatment”*) on a scale from 1 (“*not at all”*) to 5 (“*totally”*). Ratings were averaged across the three items. Last, after all treatment sessions, therapists were asked the open ended question ‘*How did you experience working with YourSkills with interactive virtual reality?*’ We used their answers to add relevant descriptions of therapists’ experiences to the data.

#### Children’s appreciation

Children rated two items on their treatment appreciation at the end of each session (i.e., “*I liked what we did today”* and “*I am looking forward to the next time”*) on a scale from 1 (“*not at all”*) to 5 (“*totally”*). Ratings were averaged between the two items.

#### Aggressive behavior

We assessed children’s aggressive behavior in the past week during each treatment session. To assess weekly change in children’s aggression during the treatment, a measure was needed that can be used for such frequently repeated measurements (Lischetzke, 2014). Therefore, we chose to use a new weekly report measure (Alsem et al., n.d.). Parents and children rated three items (i.e., “*This week I/my child fought with someone,”* “*This week I/my child kicked or beat someone,”* and “*This week I/my child called someone names”*) on a scale from 1 (“*never”*) to 5 (“*very often”*). Ratings were averaged across items. The child report version of this measure was investigated in another study (*n* = 223, *M*_age_ = 10.18, *SD* = 1.21; Alsem et al., n.d.). Results showed adequate internal consistency (i.e., Cronbach’s α’s ranged from .64 to .69 over 4 weeks) and supported the convergent and concurrent validity.

### Analyses

We used descriptive statistics to investigate treatment feasibility and children’s appreciation. To explore whether children’s aggression decreased over the treatment period, we inspected means and standard deviations (SD) of aggression in week 1 and week 10 for child and parent report. We also evaluated graphs of within-person change in aggression by plotting all 10 ratings over the treatment period. As our small-sample study was not designed to examine significant changes over time at the group level, no statistical tests were conducted.

We had missing data on parent reported aggression for two children because one parent did not speak Dutch and the other parent forgot to provide ratings for more than half of the weeks. For missing data of aggression being used in the graphs, we used last observation carried forward (LOCF). This approach seemed appropriate as no participants dropped out of the study and the percentage of missing data was low (2%).

## Results

### Feasibility

Therapists indicated that working with the interactive virtual reality was feasible. Despite working with new equipment, they managed to carry out almost all session elements (*M* = 98%; range = 95–100% across therapists and sessions). In the open evaluation question, therapists indicated that in the first sessions they needed extra preparation time (15 minutes) to set up the virtual reality equipment. However, after a few sessions, they were able to set it up quickly and easily.

Therapists experienced few technical problems. They effectively used the virtual reality equipment in 59 of the 60 sessions (98.3%). Only in one session did a therapist encounter a technical problem (i.e., the VR headset stopped tracking) that could not be solved before starting the session, upon which she decided to use role plays instead. In eight other sessions, therapists encountered a small technical problem while setting up the equipment, such as hearing no sound through the headset or not seeing the controllers in the virtual reality. They resolved these small problems themselves.

Therapists indicated that children practiced more in virtual reality than the recommended 10 minutes (*M* = 11.7 minutes and *SD* = 1.35). Within this practice time, children practiced their new skill more often than the recommended two times (*M* = 3.4 and *SD* = 1.08). The last two sessions were meant for skill rehearsal. In these sessions, children practiced even longer (i.e., *M* = 13.0 minutes and *SD* = 2.74 in session 9; *M* = 14.2 and *SD* = 2.04 in session 10).

Therapists were satisfied with how they delivered the treatment (*M* = 4.06 and *SD* = 0.49, on a 5-point scale). In response to the open evaluation question, therapists indicated that they had an overall positive experience using the virtual reality. They experienced that skill rehearsal in virtual reality is important to reinforce children’s learned skills. In addition, they indicated that children participated very actively in the virtual environment and quickly knew how to use the virtual reality equipment.

### Children’s appreciation

Children liked the treatment very much. Across the ten sessions, children’s average appreciation score was 4.68 (*SD* = 0.33) on a 5-point scale. Looking at the treatment sessions separately, we found that children were positive about all ten sessions (i.e., mean appreciation scores ranged from 4.44 to 4.83 across sessions).

### Changes in aggressive behavior

As predicted, parent reported aggression decreased between week 1 (*M* = 3.25 and *SD* = 0.88) and week 10 (*M* = 2.08 and *SD* = 0.57). This is an average decrease of 1.17 (*SD* = 0.58), equaling 2.02 *SD*s improvement. However, when looking at child reported aggression, almost no change was seen between week 1 (*M* = 2.06 and *SD* = 0.53) and week 10 (*M* = 1.78 and *SD* = 0.60) possibly because children’s aggression ratings were already modest at pretest (i.e., *M* = 2.06 on a scale of 1–5).

**Figure 2.**
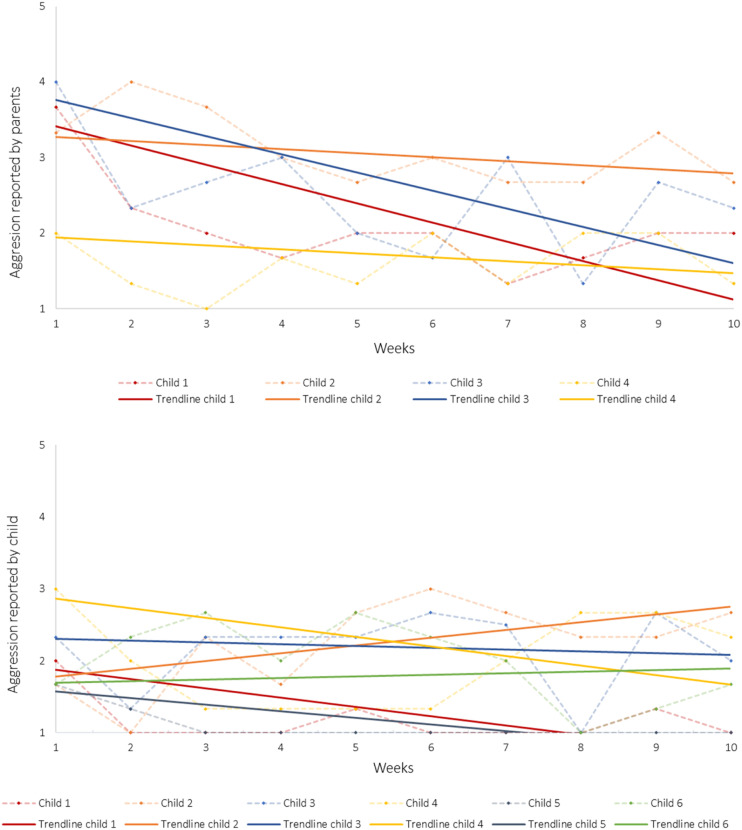
Aggressive behavior reported by parents and children over the weeks. *Note*. Dotted lines reflect reported scores and solid lines reflect linear trendlines.

Next, we created graphs to plot within-person change in aggression over the ten weeks, both for parent and child ratings (Figure 2). These graphs show a similar pattern of decreasing aggression levels according to parents and stable modest levels of aggression according to children. For child reported aggression, small increases were observed for Child 2 and Child 6. We calculated reliable change indices (RCI) to further interpret these findings, although it should be noted these RCIs are based on statistics of only six children (Table A1 in Appendix A). For Child 6, the RCI suggests no reliable change. For Child 2, the RCI suggests moderate deterioration, which is in contrast with the RCI for parent report, suggesting recovery. Thus, there was no clear indication of increases of aggressive behavior across multiple sources of information for any participant.

## Discussion

The aim of the present study was to evaluate the feasibility of a newly developed CBT with interactive virtual reality in a sample of six boys with aggressive behavior problems. Results were promising: therapists indicated that providing the treatment was feasible. They were able to complete almost all session elements, deliver the recommended amount of practice time in virtual reality, and they experienced few technical issues. Therapists were generally satisfied with their delivery of the treatment. Children highly appreciated the treatment, which was also recognized by therapists, who indicated that children actively participated in the virtual environment. Given that the main aim of our study was to examine feasibility rather than effectiveness, we only explored possible change in aggression. Parents reported decreases in children’s aggressive behavior, however, children reported low levels of aggression at pretest that did not decrease. Although this was a feasibility study with a small sample, it does give a preliminary indication that using virtual reality to treat boys from 8 to 12 years with aggressive behavior problems is feasible and may potentially reduce aggressive behavior.

Interactive virtual reality may be a promising tool to enhance CBT for children with aggressive behavior problems. It allows children to repeatedly practice social interactions in an individually tailored way, without the risks inherent in group treatment (Dodge et al., 2006). Moreover, it provides an emotionally involving practice environment, where children can frequently rehearse regulation skills in realistic anger-provoking social situations. Future virtual reality treatments could also include recordings of children’s successful behavior in the virtual reality situations and use this to stimulate social confirmation by showing the recordings to parents. Last but not least, our study showed that interactive virtual reality treatment is an appealing method for children with aggressive behavior problems, which may prevent motivation problems and resistance to therapy, as often encountered in this population (Frick, 2012; Lochman et al., 2019).

### Strengths and limitations

A strength of this study was that we examined children in routine care at clinical institutions. Children were referred to these institutions for their aggressive behavior problems, but none of the participants or their parents actively sought this new form of treatment. All children completed the treatment and reported high levels of appreciation, and all therapists were satisfied with how they were able to deliver the treatment. This indicates that using interactive virtual reality is feasible and acceptable for children in routine care.

This study also had its limitations. First, the small sample size warrants cautious interpretation of the results. The present findings should be seen as indicative of the potential of virtual reality in CBT for children with aggressive behavior problems rather than as conclusive evidence. Second, because the study lacked a control condition and included a very small sample, it is uncertain whether changes in aggressive behavior were an actual effect of the treatment. Hence, we could only explore within-person changes to provide a preliminary indication of whether it would be promising to conduct a large efficacy trial.

Given the feasibility and high appreciation of the treatment, and the preliminary findings for aggressive behavior, a next logical step would be to test the positive indications from this study rigorously in a randomized controlled trial. Such a controlled trial could also be used to determine whether virtual reality actually *enhances* CBT—that is, whether CBT with interactive virtual reality is more effective than the same CBT without virtual reality. The added value of interactive virtual reality may thus be established in order to determine whether its effects outweigh the financial costs of implementing this tool. Such future research is important, especially since the use of virtual reality seems theoretically promising and is increasingly called for by practitioners (Lindner et al., 2019).

### Conclusions

In conclusion, we developed a new CBT with interactive virtual reality for boys with aggressive behavior problems using an emotionally engaging and individually tailored practice environment. The present study showed that this interactive virtual reality treatment is feasible and appealing to children and therapists and has the potential to reduce aggressive behavior. These findings suggest enough promise to conduct randomized controlled trials needed to determine whether CBT with interactive virtual reality reduces aggressive behavior more strongly and for more children than regular CBT would.

## Appendix A

The reliable change index (RCI) indicates whether an individual change score is significantly larger than would be expected by chance (Jacobson & Truax, 1991). We calculated RCIs based on children’s difference scores between week 1 and week 10, the standard error, and scale reliability in week 1. To obtain insight in the clinical significance of the RCIs, we interpreted them according to the guidelines of Wise (2004). The RCIs should be interpreted with caution because they are based on standard deviations and reliability statistics of only six children.

**Table A1.** Reliable change indices with clinical significance terms for aggression from pretreatment (week 1) to posttreatment (week 10).

Child	Parent reported aggression		Child reported aggression	
1	5.04	Recovered	1.64	Remitted
2	2.01	Recovered	−1.64	Moderately deteriorated
3	5.04	Recovered	0.55	No change
4	2.01	Recovered	1.10	Improved
5	—	—	1.10	Improved
6	—	—	0.00	No change

*Note*. For two parents, no reliable change index was computed due to missing scores in week 10.

## References

[bibr1-13591045211026160] Alsem S. C. Keulen J. Verhulp E. E. van Dijk A. De Castro B. O. (n.d.). Capturing mechanisms of change: Weekly covariation in anger regulation, hostile intent attribution, and children’s aggression. Manuscript Under Review. 10.1002/ab.22019 PMC9306713 35049063

[bibr2-13591045211026160] Burkey M. D. Hosein M. Morton I. Purgato M. Adi A. Kurzrok M. Kohrt B. A. Tol W. A. (2018). Psychosocial interventions for disruptive behaviour problems in children in low- and middle-income countries: A systematic review and meta-analysis. Journal of Child Psychology and Psychiatry, 59(9), 982-993. doi:10.1111/jcpp.12894 29633271

[bibr3-13591045211026160] Chorpita B. F. Daleiden E. L. (2009). Mapping evidence-based treatments for children and adolescents: Application of the distillation and matching model to 615 treatments from 322 randomized trials. Journal of Consulting and Clinical Psychology, 77(3), 566-579. doi:10.1037/a0014565 19485596

[bibr4-13591045211026160] Crick N. R. Dodge K. A. (1994). A review and reformulation of social information-processing mechanisms in children’s social adjustment. Psychological Bulletin, 115(1), 74-101. doi:10.1037/0033-2909.115.1.74

[bibr5-13591045211026160] De Mooij B. Fekkes M. Scholte R. H. J. Overbeek G. (2020). Effective components of social skills training programs for children and adolescents in nonclinical samples: A multilevel meta-analysis. Clinical Child and Family Psychology Review, 23(2), 250-264. DOI:10.1007/s10567-019-00308-x. 31919684

[bibr6-13591045211026160] Dodge K. A. (1990). The structure and function of reactive and proactive aggression. In Pepler D. Rubin K. H. (Eds.), The development and treatment of childhood aggression (pp. 201-218). Erlbaum.

[bibr7-13591045211026160] Dodge K. A. Dishion T. J. Lansford J. E. (2006). Deviant peer influences in intervention and public policy for youth. Social Policy Report, 20(1), 1-20. doi:10.1002/j.2379-3988.2006.tb00046.x

[bibr8-13591045211026160] Frick P. J. (2012). Developmental pathways to conduct disorder: Implications for future directions in research, assessment, and treatment. Journal of Clinical Child & Adolescent Psychology, 41(3), 378-389. doi:10.1080/15374416.2012.664815 22475202

[bibr9-13591045211026160] Garland A. F. Hawley K. M. Brookman-Frazee L. Hurlburt M. S. (2008). Identifying common elements of evidence-based psychosocial treatments for children’s disruptive behavior problems. Journal of the American Academy of Child and Adolescent Psychiatry, 47(5), 505-514. doi:10.1097/CHI.0b013e31816765c2. 18356768

[bibr10-13591045211026160] Hadley W. Houck C. Brown L. K. Spitalnick J. S. Ferrer M. Barker D. (2019). Moving beyond role-play: Evaluating the use of virtual reality to teach emotion regulation for the prevention of adolescent risk behavior within a randomized pilot trial. Journal of Pediatric Psychology, 44(4), 425-435. doi:10.1093/jpepsy/jsy092 30551157 PMC6481385

[bibr11-13591045211026160] Jacobson N. S., Truax P. (1991). Clinical significance: A statistical approach to defining meaningful change in psychotherapy research. Journal of Consulting and Clinical Psychology, 59(1), 12-19. doi:10.1037/0022-006X.59.1.12 2002127

[bibr12-13591045211026160] Kandalaft M. R. Didehbani N. Krawczyk D. C. Allen T. T. Chapman S. B. (2013). Virtual reality social cognition training for young adults with high-functioning autism. Journal of Autism and Developmental Disorders, 43(1), 34-44. doi:10.1007/s10803-012-1544-6 22570145 PMC3536992

[bibr13-13591045211026160] Landenberger N. A., Lipsey M. W. (2005). The positive effects of cognitive-behavioral programs for offenders: A meta-analysis of factors associated with effective treatment. Journal of Experimental Criminology, 1(4), 451-476. doi:10.1007/s11292-005-3541-7

[bibr14-13591045211026160] Lindner P. Miloff A. Zetterlund E. Reuterskiöld L. Andersson G. Carlbring P. (2019). Attitudes toward and familiarity with virtual reality therapy among practicing cognitive behavior therapists: A cross-sectional survey study in the era of consumer VR platforms. Frontiers in Psychology, 10, 1-10. doi:10.3389/fpsyg.2019.00176 30800086 PMC6376952

[bibr15-13591045211026160] Lischetzke T. (2014). Daily diary methodology. In Michalos A. C. (Ed.), Encyclopedia of quality of life and well-being research (pp. 1413-1419). Springer.

[bibr16-13591045211026160] Lochman J. E. Boxmeyer C. L. Jones S. Qu L. Ewoldsen D. Nelson W. M. (2017a). Testing the feasibility of a briefer school-based preventive intervention with aggressive children: A hybrid intervention with face-to-face and internet components. Journal of School Psychology, 62, 33-50. DOI:10.1016/j.jsp.2017.03.010. 28646974 PMC5492991

[bibr17-13591045211026160] Lochman J. E. Boxmeyer C. L. Kassing F. L. Powell N. P. Stromeyer S. L. (2019). Cognitive behavioral intervention for youth at risk for conduct problems: Future directions. Journal of Clinical Child & Adolescent Psychology, 48(5), 799-810. doi:10.1080/15374416.2019.1567349 30892949 PMC6710135

[bibr18-13591045211026160] Lochman J. E. Dishion T. J. Powell N. P. Boxmeyer C. L. Qu L. Sallee M. (2015). Evidence-based preventive intervention for preadolescent aggressive children: One-year outcomes following randomization to group versus individual delivery. Journal of Consulting and Clinical Psychology, 83(4), 728-735. doi:10.1037/ccp0000030 26098373 PMC4516613

[bibr19-13591045211026160] Lochman J. E. Kassing F. Sallee M. Stromeyer S. L. (2017b). Factors influencing intervention delivery and outcomes. In The Wiley handbook of disruptive and impulse-control disorders (pp. 485-500). John Wiley & Sons, Ltd.

[bibr20-13591045211026160] Lochman J. E., Matthys W. (2017). The Wiley handbook of disruptive and impulse-control disorders. John Wiley & Sons, Ltd.

[bibr21-13591045211026160] Lochman J. E. Wells K. C. Lenhart L. A. (2008). Coping power cild group program. Oxford University Press, Inc.

[bibr22-13591045211026160] Loeber R., Farrington D. P. (2000). Young children who commit crime: Epidemiology, developmental origins, risk factors, early interventions, and policy implications. Development and Psychopathology, 12(4), 737-762. doi:10.1017/S0954579400004107 11202042

[bibr23-13591045211026160] Matthys W. Maassen G. H. Cuperus J. M. Van Engeland H. (2001). The Assessment of the situational specificity of children’s problem behaviour in peer-peer context. Journal of Child Psychology and Psychiatry, 42(3), 413-420. doi:10.1017/S002196300100703X 11321210

[bibr24-13591045211026160] McCart M. R. Priester P. E. Davies W. H. Azen R. (2006). Differential effectiveness of behavioral parent-training and cognitive-behavioral therapy for antisocial youth: A meta-analysis. Journal of Abnormal Child Psychology, 34(4), 527-543. DOI:10.1007/s10802-006-9031-1. 16838122

[bibr25-13591045211026160] Menting A. T. A. Albrecht G. De Castro B. O. (2015). Effective elementen van interventies tegen externaliserende gedagsproblemen bij jeugd [Effective elements of interventions for externalizing problem behavior in youth]. Utrecht University.

[bibr26-13591045211026160] Newbutt N. Sung C. Kuo H.-J. Leahy M. J. Lin C.-C. Tong B. (2016). Brief report: A pilot study of the use of a virtual reality headset in autism populations. Journal of Autism and Developmental Disorders, 46(9), 3166-3176. doi:10.1007/s10803-016-2830-5 27272115

[bibr27-13591045211026160] Park K.-M. Ku J. Choi S.-H. Jang H.-J. Park J.-Y. Kim S. I. Kim J.-J. (2011). A virtual reality application in role-plays of social skills training for schizophrenia: A randomized, controlled trial. Psychiatry Research, 189(2), 166-172. doi:10.1016/j.psychres.2011.04.003 21529970

[bibr28-13591045211026160] Saiano M. Pellegrino L. Casadio M. Summa S. Garbarino E. Rossi V. Dall’Agata D. Sanguineti V. (2015). Natural interfaces and virtual environments for the acquisition of street crossing and path following skills in adults with autism spectrum disorders: A feasibility study. Journal of NeuroEngineering and Rehabilitation, 12(1), 1-13. DOI:10.1186/s12984-015-0010-z. 25885279 PMC4344805

[bibr29-13591045211026160] Sukhodolsky D. G. Smith S. D. McCauley S. A. Ibrahim K. Piasecka J. B. (2016). Behavioral interventions for anger, irritability, and aggression in children and adolescents. Journal of Child and Adolescent Psychopharmacology, 26(1), 58-64. doi:10.1089/cap.2015.0120 26745682 PMC4808268

[bibr30-13591045211026160] Suveg C. Southam-Gerow M. A. Goodman K. L. Kendall P. C. (2007). The role of emotion theory and research in child therapy development. Clinical Psychology: Science and Practice, 14(4), 358-371. doi:10.1111/j.1468-2850.2007.00096.x

[bibr31-13591045211026160] Underwood M. K. (2002). Sticks and stones and social exclusion: Aggression among girls and boys. In Smith P. K. Hart C. H. (Eds.), Blackwell handbook of childhood social development (pp. 533-548). Blackwell Publishers Ltd. doi:10.1111/b.9780631217534.2004.x

[bibr32-13591045211026160] Van Manen T. G. (2001). Zelfcontrole [Self-Control]. Bohn Stafleu Van Loghum.

[bibr33-13591045211026160] Verhoef R. E. J. Van Dijk A. Verhulp E. E. De Castro B. O. (2021). Interactive virtual reality assessment of aggressive social information processing in boys with behavior problems: A pilot study. Clinical Psychology & Psychotherapy, (Advance online publication). DOI:10.1002/cpp.2620. PMC8361679 34048619

[bibr34-13591045211026160] Vitaro F. Brendgen M. Tremblay R. E. (2002). Reactively and proactively aggressive children: Antecedent and subsequent characteristics. Journal of Child Psychology and Psychiatry, 43(4), 495-505. doi:10.1111/1469-7610.00040 12030595

[bibr35-13591045211026160] Weisz J. R., Kazdin A. E. (2017). Evidence-based psychotherapies for children and adolescents. Guilford Publications.

[bibr36-13591045211026160] Weisz J. R. Kuppens S. Ng M. Y. Vaughn-Coaxum R. A. Ugueto A. M. Eckshtain D. Corteselli K. A. (2019). Are psychotherapies for young people growing stronger? Tracking trends over time for youth anxiety, depression, attention-deficit/hyperactivity disorder, and conduct problems. Perspectives on Psychological Science, 14(2), 216-237. doi:10.1177/1745691618805436 30571478

[bibr37-13591045211026160] Wilson S. J., Lipsey M. W. (2007). School-based interventions for aggressive and disruptive behavior: Update of a meta-analysis. American Journal of Preventive Medicine, 33(2), S130-S143. DOI:10.1016/j.amepre.2007.04.011. 17675014 PMC2246021

[bibr38-13591045211026160] Wise E. A. (2004). Methods for analyzing psychotherapy outcomes: A review of clinical significance, reliable change, and recommendations for future directions. Journal of Personality Assessment, 82(1), 50-59. doi:10.1207/s15327752jpa8201_10 14979834

